# New mechanisms and biomarkers of lymph node metastasis in cervical cancer: reflections from plasma proteomics

**DOI:** 10.1186/s12014-023-09427-8

**Published:** 2023-09-09

**Authors:** Sai Han, Xiaoli Liu, Shuang Ju, Wendi Mu, Gulijinaiti Abulikemu, Qianwei Zhen, Jiaqi Yang, Jingjing Zhang, Yi Li, Hongli Liu, Qian Chen, Baoxia Cui, Shuxia Wu, Youzhong Zhang

**Affiliations:** 1 Department of Obstetrics and Gynecology, Qilu Hospital of Shandong University, 107 Wenhua Xi Road, Jinan, Shandong 250012 People’s Republic of China; 2 Department of Obstetrics and Gynecology, the Fifth People’s Hospital of Jinan, Jinan, Shandong 250012 People’s Republic of China

**Keywords:** DIA, Cervical cancer, LVSI, LNM, Biomarker

## Abstract

**Objective:**

Lymph node metastasis (LNM) and lymphatic vasculature space infiltration (LVSI) in cervical cancer patients indicate a poor prognosis, but satisfactory methods for diagnosing these phenotypes are lacking. This study aimed to find new effective plasma biomarkers of LNM and LVSI as well as possible mechanisms underlying LNM and LVSI through data-independent acquisition (DIA) proteome sequencing.

**Methods:**

A total of 20 cervical cancer plasma samples, including 7 LNM-/LVSI-(NC), 4 LNM-/LVSI + (LVSI) and 9 LNM + /LVSI + (LNM) samples from a cohort, were subjected to DIA to identify differentially expressed proteins (DEPs) for LVSI and LNM. Subsequently, Gene Ontology (GO) and Kyoto Encyclopedia of Genes and Genomes (KEGG) analyses were performed for DEP functional annotation. Protein–protein interaction (PPI) and weighted gene coexpression network analysis (WGCNA) were used to detect new effective plasma biomarkers and possible mechanisms.

**Results:**

A total of 79 DEPs were identified in the cohort. GO and KEGG analyses showed that DEPs were mainly enriched in the complement and coagulation pathway, lipid and atherosclerosis pathway, HIF-1 signal transduction pathway and phagosome and autophagy. WGCNA showed that the enrichment of the green module differed greatly between groups. Six interesting core DEPs (SPARC, HPX, VCAM1, TFRC, ERN1 and APMAP) were confirmed to be potential plasma diagnostic markers for LVSI and LNM in cervical cancer patients.

**Conclusion:**

Proteomic signatures developed in this study reflected the potential plasma diagnostic markers and new possible pathogenesis mechanisms in the LVSI and LNM of cervical cancer.

**Supplementary Information:**

The online version contains supplementary material available at 10.1186/s12014-023-09427-8.

## Introduction

Cervical cancer is one of the most common malignant gynaecological tumours. It is also one of the leading causes of morbidity and mortality among females worldwide [1]. The prognosis of cervical cancer patients is highly related to postoperative pathological factors. Lymph node metastasis (LNM) is a high-risk factor, and lymphatic vasculature space infiltration (LVSI) is a moderate-risk factor. Given this, the International Federation of Gynaecology & Obstetrics (FIGO) staging system underwent revision in 2018 [2]. The new staging system expanded the stage III classifications to include stage IIIC: All cases with lymph node involvement were classified as stage IIIC; those with pelvic or para-aortic lymph node involvement were classified as stage IIIC1 or stage IIIC2. The classification was also based on whether diagnosis was made radiographic assessment only (r) or pathological confirmation (p). The stage was classified as IIIC1r if imaging showed pelvic lymph node metastasis. If confirmed by pathological results, the stage was IIIC1p. However, the latest meta-analysis showed that imaging examinations are barely satisfactory. The false-negative rate of PET-CT and MRI or CT imaging for the detection of para-aortic lymph node metastasis reaches 11–21%. The sensitivity of transvaginal ultrasonography (TVUS) in diagnosing LNM in cervical cancer is only 52% [3, 4]. Hence, there is a need to identify other modalities with better sensitivity and specificity to act as effective screening and diagnostic tools for the diagnosis of LNM in cervical cancer.

Serological detection of squamous cell carcinoma antigen (SCC-Ag) is currently widely used for advanced cervical cancer. Some studies have shown that SCC-Ag is related to recurrence and survival but cannot be used as an indicator of lymph node metastases [5–7]. At present, data-independent acquisition (DIA)-based quantitative proteomics technology can be used for protein identification and quantification. Compared with traditional data-dependent acquisition (DDA) mass spectrometry technology, DIA performed the full scanning range of the mass spectrum, and all ions are detected and crushed. Thus, the information of all ions in the sample can be obtained without omission or difference. It has been applied in biomarker screening, fundamental research, and other fields [8–10]. It has many advantages, such as high accuracy, deep proteome coverage and high quantitative reproducibility. In this study, we performed DIA to provide full-scale protein profiles from plasma to further reveal the pathogenesis process of LVSI and LNM in cervical cancer and to reveal candidate biomarkers.

## Materials and methods

### Study design and collection of clinical samples

**Fig. 1:**
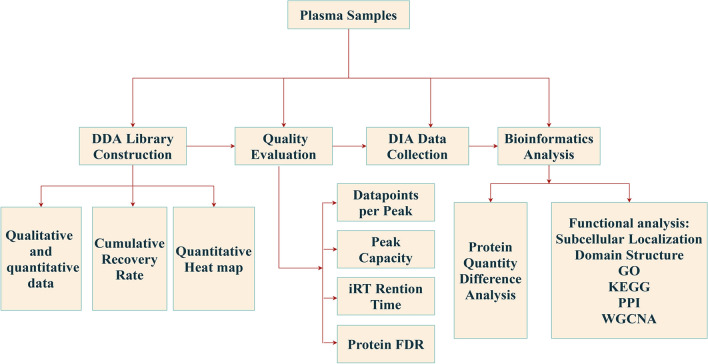
Schematic workflow of the study

The study design is shown in Fig. 1. In this study, the main inclusion criteria were as follows: (1) Diagnosis of cervical cancer by two independent pathologists; (2) No surgery or neoadjuvant chemotherapy was performed prior to plasma collection. Exclusion criteria: (1) The patients had other primary tumours; (2) The postoperative pathology showed no tumour cells. Finally, a total of 25 cervical cancer plasma samples, including LNM−/LVSI− (named NC), LNM−/LVSI + (named LVSI) and LNM + /LVSI + (named LNM) samples, were collected from the Department of Obstetrics and Gynaecology, Qilu Hospital of Shandong University from January 2019 to June 2020. 5 cervical cancer patients were excluded. 1 patient was excluded for she had both cervical and lung cancer, 4 patients were excluded due to the neoadjuvant chemotherapy before we collected the plasma samples. Among the other 20 specimens, 7 NC, 4 LVSI and 9 LNM plasma samples were analysed by DIA to investigate the protein changes in these three states.

For plasma collection, blood specimens were immediately handled according to the protocol suggested by the HUPO Plasma Proteome Project [8], and informed consent was obtained from all patients. The study was approved by the ethics committee of the Qilu Hospital of Shandong University (Ethics number: KYLL-2017–560).

### Sample preparation and fractionation for DDA library generation

The plasma pools were separated from the most abundant proteins using a Human 14 Multiple Affinity Removal System Column following the manufacturer’s protocol (Agilent Technologies). The remaining steps were the same as in a previous report [9].

### Data-dependent acquisition (DDA) mass spectrometry assay

All fractions for DDA library generation were analysed by a Thermo Scientific QExactive HF X mass spectrometer connected to an Easy nLC 1200 chromatography system (Thermo Scientific). The peptide (1.5 μg) was first loaded onto an EASY-SprayTMC18 Trapcolumn (Thermo Scientific, P/N 164946, 3 µm, 75 um*2 cm), then separated on an EASY-SprayTM C18 LC Analytical Column (Thermo Scientific, ES802, 2 µm, 75 um*25 cm) with a linear gradient of buffer B (84% acetonitrile and 0.1% Formic acid) at a flow rate of 250 nl/min over 120 min. MS detection method was positive ion, the scan range was 300–1800 m/z, the resolution for the MS1 scan was 60,000 at 200 m/z, the target automatic gain control (AGC) was 3e6, the maximum IT was 25 ms, and the dynamic exclusion parameter was 30.0 s. Each full MS–SIM scan followed 20 ddMS2 scans. The resolution for the MS2 scan was 15,000, the AGC target was 5e4, the maximum IT was 25 ms, and the normalized collision energy was 30 eV.

### Mass spectrometry assay for DIA

The peptides from each sample were analysed by LC‒MS/MS operating in the DIA mode by Shanghai Applied Protein Technology Co., Ltd. Each DIA cycle contained one full MS–SIM scan, and 30 DIA scans covered a mass range of 350–1800 m/z with the following settings: SIM full scan resolution, 120,000 at 200 m/z; AGC, 3e6; maximum IT, 50 ms; profile mode, DIA scan parameters were set as follows: resolution, 15,000; AGC target, 3e6; max IT, auto; and normalized collision energy, 30 eV. The run time was 120 min with a linear gradient of buffer B (84% acetonitrile and 0.1% formic acid) at a flow rate of 250 nl/min. QC samples (pooled sample from an equal aliquot of each sample in the experiment) were injected in DIA mode at the beginning of the MS study and after every 6 injections throughout the experiment, which was used to monitor the MS performance.

### Mass spectrometry data analysis

For DDA library data, the FASTA sequence database was searched with Spectronaut TM14.4.200727.47784 software. The database was the UniProt human database, and the iRT peptide sequence was added (Biognosys|iRTKit|). DIA data were analysed with Spectronaut TM14.4.200727.47784 by searching the above constructed spectral library. The main software parameters were set as follows: retention time prediction type was dynamic iRT, interference on MS2 level correction was enabled, and cross-run normalization was enabled. All results were filtered based on a Q value cut-off of 0.01 (equivalent to FDR < 1%).

### Bioinformatic and statistical analysis

#### Basic bioinformatics analysis

The subcellular localization, domain annotation, GO annotation, KEGG annotation, enrichment analysis, and protein‒protein interaction (PPI) analysis steps were the same as those in a previous report [10].

#### Weighted gene coexpression network analysis (WGCNA)

The WGCNA package in R (Version 1.69) was used to identify distinct protein modules among all identified proteins. A weighted protein coexpression network was generated using the log2 protein abundance sample matrix.

#### Statistical analysis

GraphPad Prism version 5.01 (GraphPad Software Inc., San Diego, CA, USA) was used for statistical analysis. In the present study, data are expressed as the means with standard deviations (SDs), and statistical comparisons were performed using Student's t test or ANOVA. P < 0.05 was considered to indicate a statistically significant result.

## Results

### Quality evaluation and identification of differentially expressed proteins (DEPs)

**Fig. 2:**
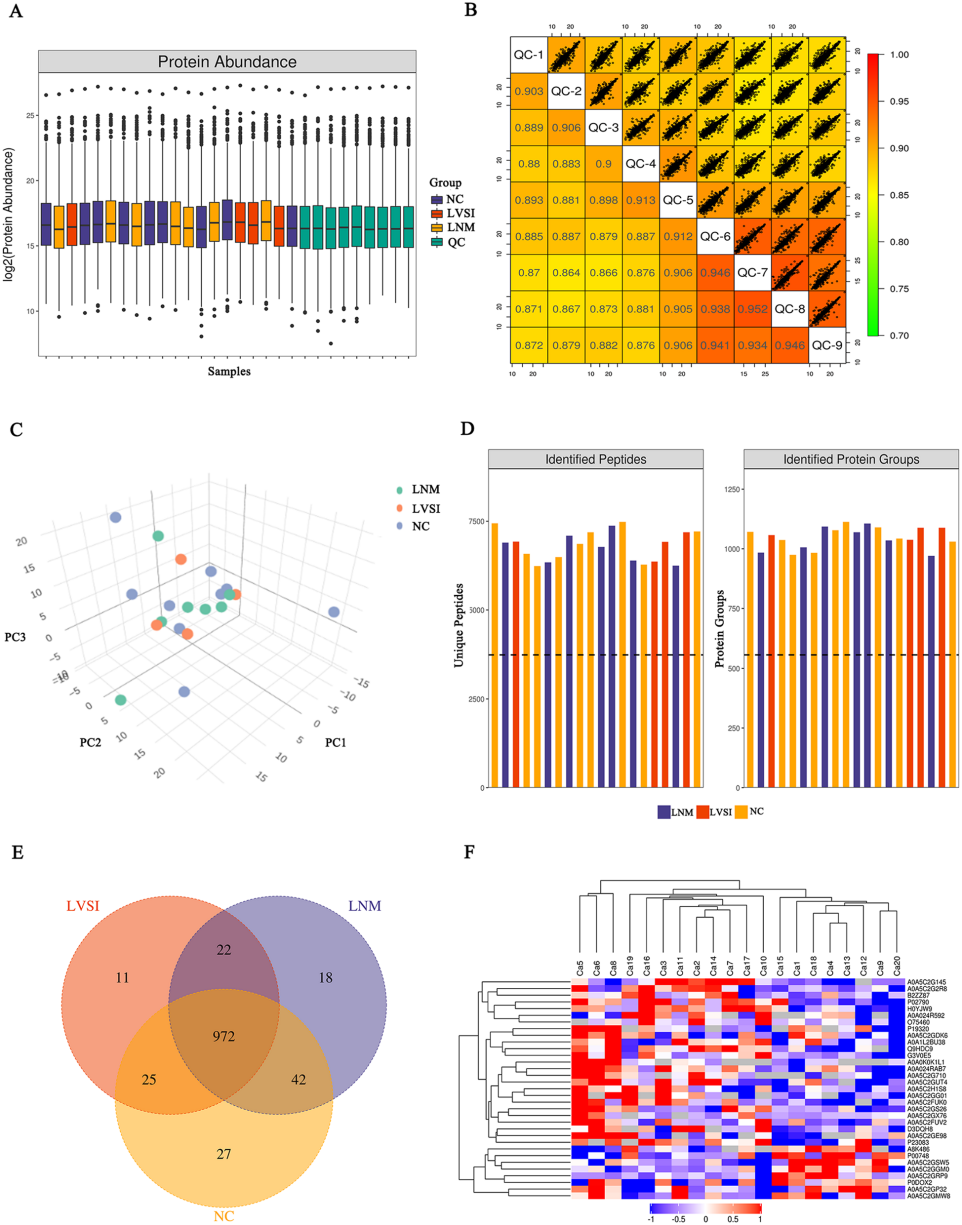
Data quality control and general overview of protein identification. **A** Protein abundance. **B** Intensity correlation of QC samples. **C** PCA score plot. Samples from the NC (n = 7), LVSI (n = 4) and LNM (n = 9) groups are plotted along the three principal components. **D** The identified peptides and proteins of the three groups. **E** Venn diagram showing the number of proteins common and unique to NC, LVSI and LNM cervical cancer patients. **F** DIA quantitative heatmap of total protein

**Fig. 3:**
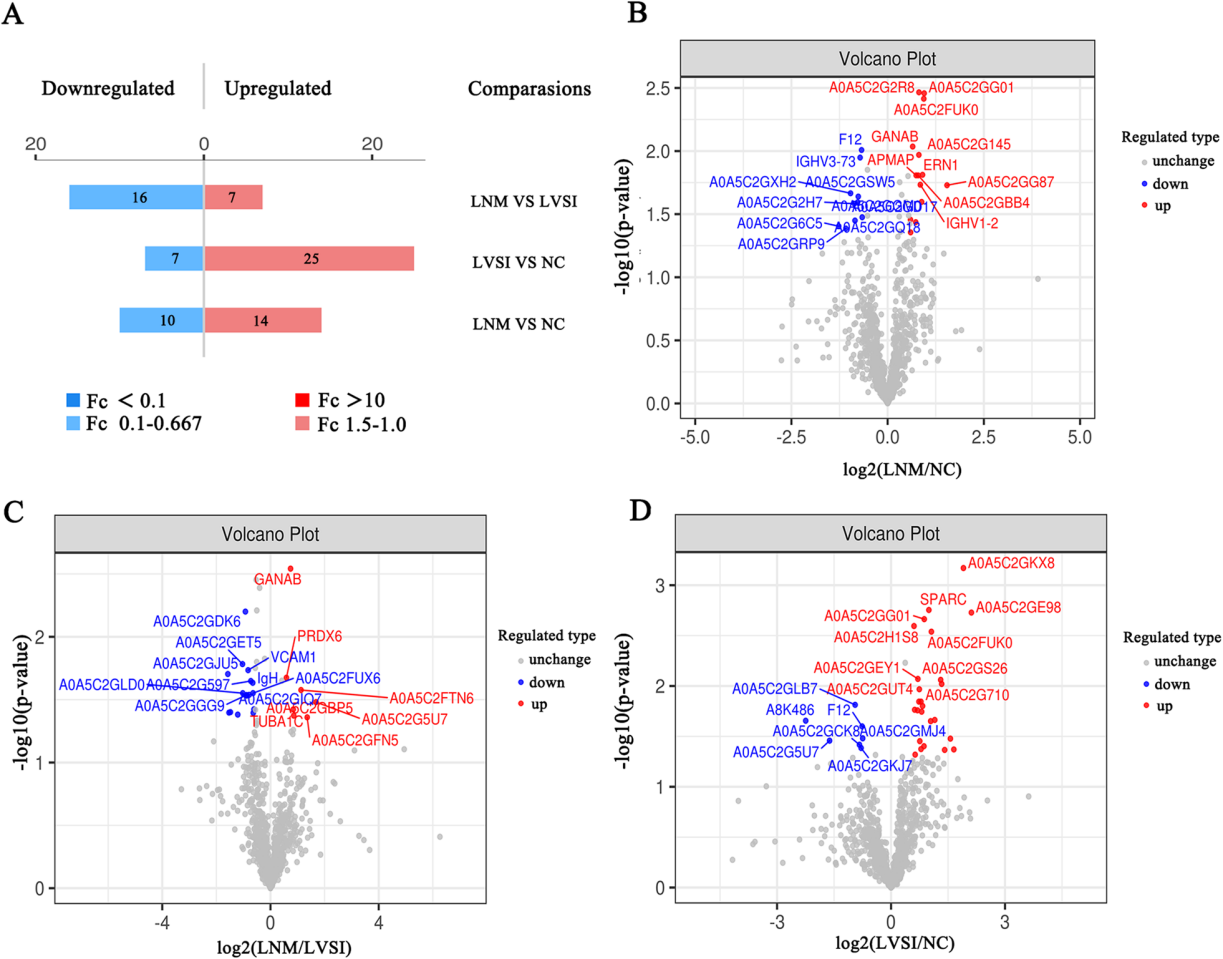
Expression of differentially expressed proteins (DEPs). **A** Bar chart of protein fold change values. **B** Volcano plot of DEPs between the LNM and NC groups. **C** Volcano plot of DEPs between the LNM and LVSI groups. **D** Volcano plot of DEPs between the LVSI and NC groups

The clinical characteristics of the 20 cervical cancer patients are summarized in Table 1. Quality control (QC) analysis was performed to evaluate the DIA proteomic information. The results suggested that there was enough protein, and the QC samples appeared to have a strong correlation (Fig. 2A, B). The principal component analysis (PCA) of the proteins showed no significant difference among these three groups (Fig. 2C). In total, 12,915 peptides associated with 1357 proteins were identified in our study (Fig. 2D). A total of 1066, 1030, and 1054 proteins were identified in NC, LVSI, and LNM, respectively. A total of 972 proteins were shared by all three groups (Fig. 2E). The DIA protein heatmap is also shown in Fig. 2F. For the three-group comparison by one-way ANOVA, proteins with a fold change (FC) ≥ 1.5 or ≤ 0.67 and P value < 0.05 were defined as DEPs. In total, 79 DEPs of the three groups were identified in this research (Fig. 3A). The LNM group had a total of 24 DEPs, including 14 upregulated proteins and 10 downregulated proteins, compared with the NC group (Fig. 3A, B; Additional file 1: Table S1). There were 23 DEPs (7 upregulated proteins and 16 downregulated proteins) in the LNM group compared with the LVSI group (Fig. 3A, C; Additional file 1: Table S1). There were 32 DEPs (25 upregulated proteins and 7 downregulated proteins) in the LVSI group compared with the NC group (Fig. 3A, D; Additional file 1: Table S1).

**Table 1:** Summary of the study cohorts

Characteristics/ Number	Age(year)	TCT	HPV type	FIGO stage	DSI	LVSI	LNM	Group
Ca1	39			IB3	> 2/3	−	−	NC
Ca2	33	−	16	IIIC1	1	+	+	LNM
Ca3	31			IIIC1	1	+	+	LNM
Ca4	68		58	IIA2	> 2/3	−	−	NC
Ca5	56	HSIL	16	IIA1	> 2/3	+	−	LVSI
Ca6	41			IB2	< 1/3	+	−	LVSI
Ca7	38	AGC	16	IIIC1	> 1/2	+	+	LNM
Ca8	47			IB2	> 2/3	+	−	LVSI
Ca9	61			IB3	> 1/2	−	−	NC
Ca10	42		16	IIIC2	1	+	+	LNM
Ca11	54	ASC-H	16/45	IB2	> 2/3	+	−	LVSI
Ca12	57		16	IB3	1	−	−	NC
Ca13	52		16	IB3	< 1/2	−	−	NC
Ca14	46	−	16/53/40	IIIC1	1	+	+	LNM
Ca15	26	HSIL	16	IIA2	1	−	−	NC
Ca16	52			IIIC1	1	+	+	LNM
Ca17	39			IB2	1	−	−	NC
Ca18	36	HSIL	31	IB2	< 1/3	−	−	NC
Ca19	52	HSIL	18	IIIC1	< 1/3	+	+	LNM
Ca20	56			IB2	> 2/3	−	−	NC

HSIL, high grade squamous intraepithelial lesion; DSI, deep interstitial infiltration; LNM, lymph node metastasis; LVSI, lymph vascular space involvement

### Functional annotation analysis

**Fig. 4:**
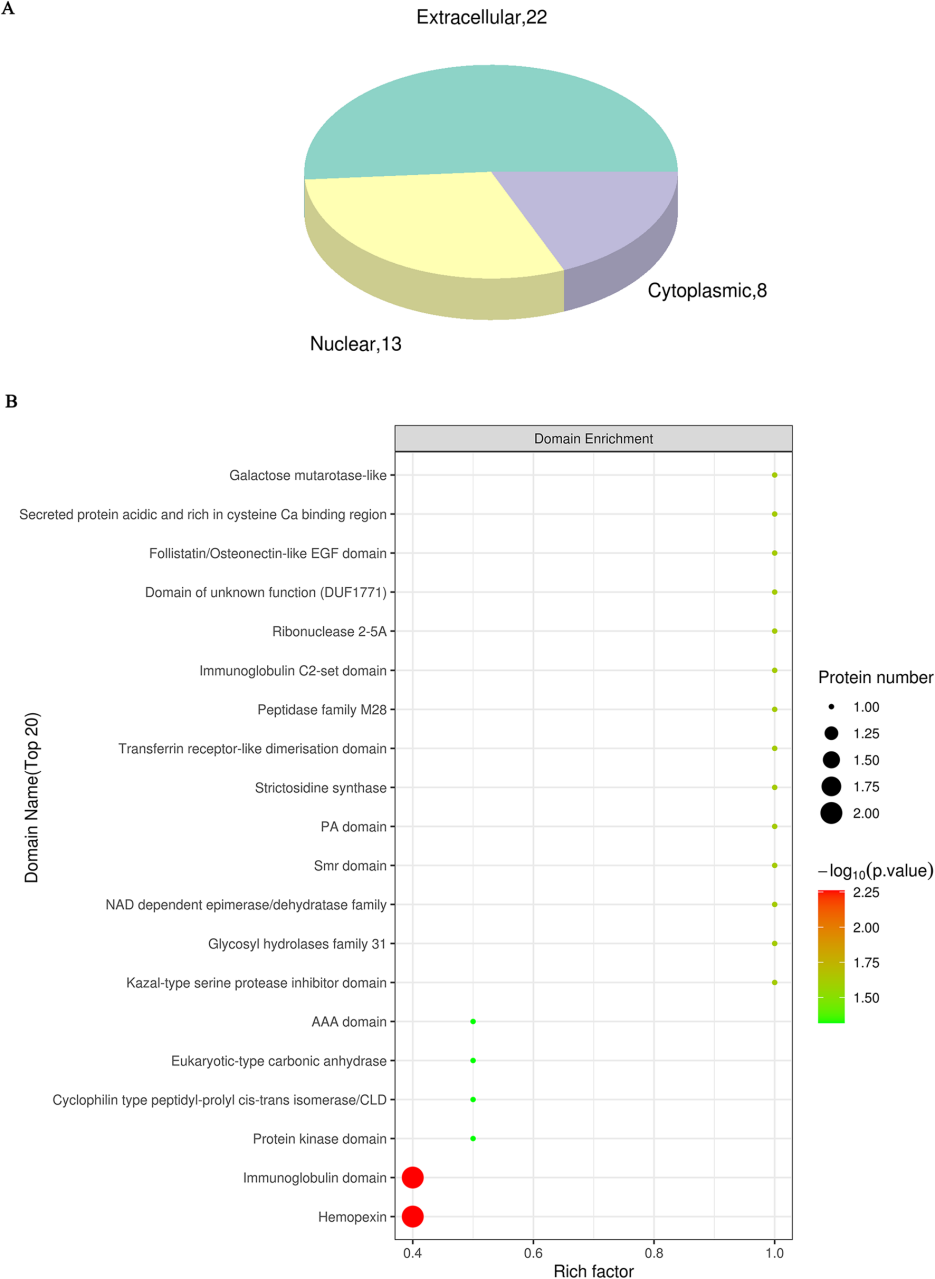
Subcellular localization and domain enrichment analysis of DEPs. **A** Pie chart of subcellular localization of DEPs. **B** Domain enrichment analysis of DEPs

**Fig. 5:**
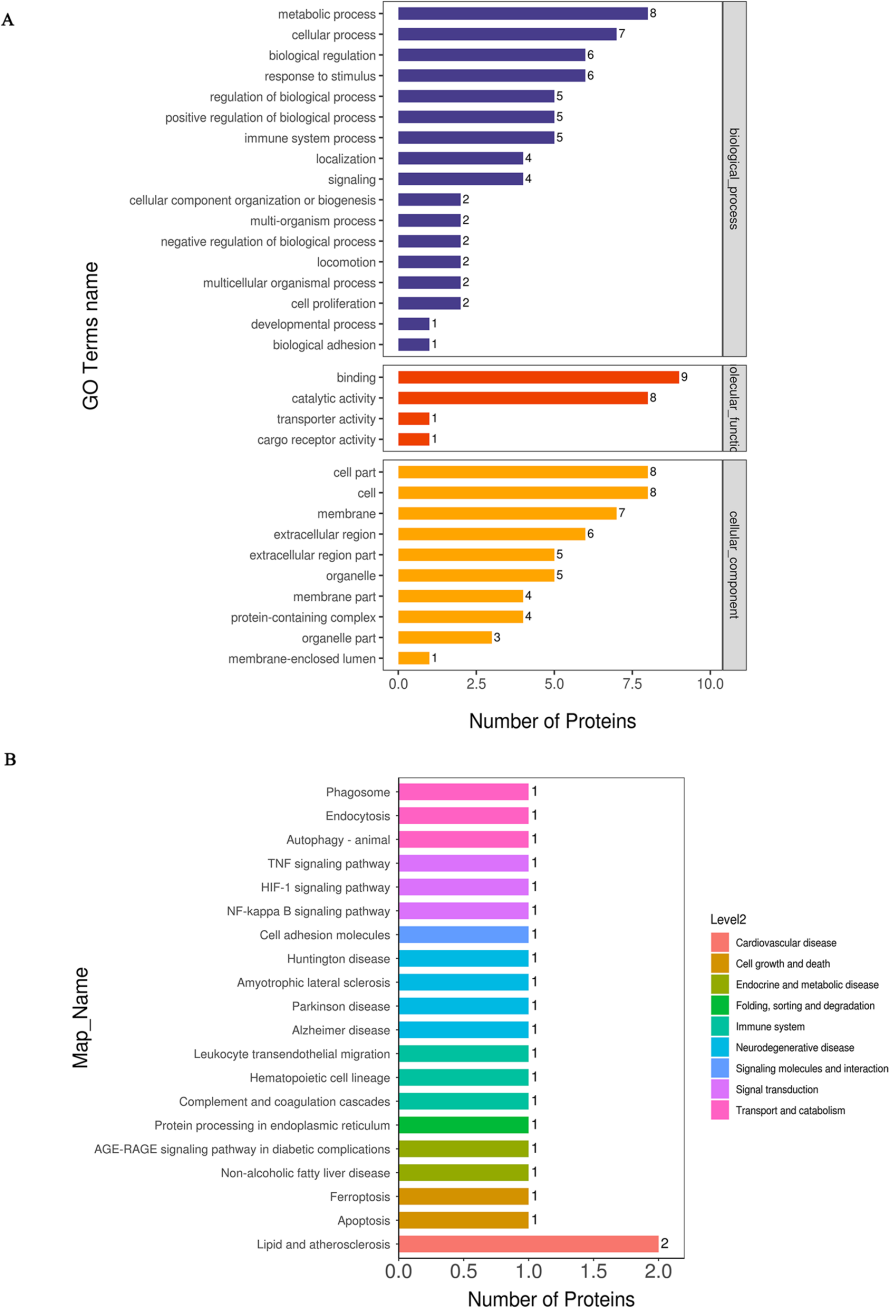
GO and KEGG pathway analysis of DEPs between the NC, LVSI and LNM groups. **A** GO analysis. The results for the biological process (BP), molecular function (MF) and cellular component (CC) categories are presented. **B** KEGG pathway analysis

To better understand the function of these DEPs, subcellular localization, domain analysis, gene ontology (GO) and Kyoto Encyclopedia of Genes and Genomes (KEGG) pathway enrichment analyses were conducted. Extracellular proteins were most abundant in the subcellular localization analysis (Fig. 4A). Domain enrichment analysis showed that haemopexin and immunoglobulin had the largest changes in both protein numbers and P values (Fig. 4B). GO analysis (Fig. 5A) showed that the DEPs between the three groups were mainly enriched in the metabolic process in the biological process (BP) category, binding in the molecular function (MF) category, and cell part on the cellular component (CC) category. Moreover, KEGG analysis revealed that the DEPs were mainly enriched in the lipid and atherosclerosis pathways, which are related to cardiovascular disease; other pathways, such as the HIF-1 signal transduction pathway, phagosome and autophagy and ferroptosis pathway, were also of interest (Fig. 5B). Notably, the results above are from ANOVA of the three groups. The results for pairwise comparisons between each pair of groups are provided in the supplementary materials (Additional file 1: Figure S1–7).

### Protein‒protein interaction (PPI) network

**Fig. 6:**
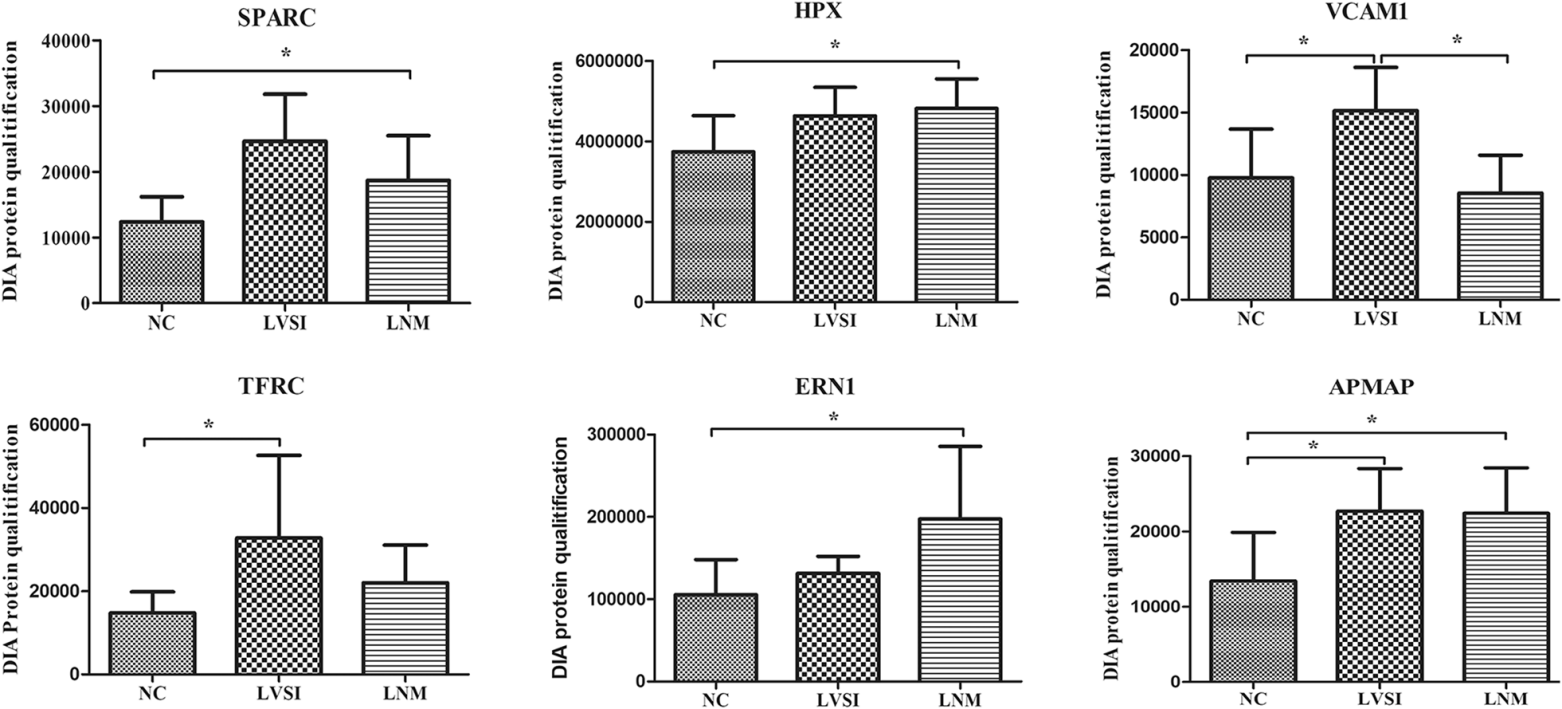
The DIA expression pattern of six hub proteins

The 79 DEPs were submitted to the STRING 11.0 database via Cytoscape 3.8.0 software to obtain the PPI network diagram. In the PPT network, the nodes represent the difference in protein expression, and the lines represent the connection degree. A high fold change and a high connectivity degree are two characteristics of hub proteins. We identified six hub proteins, which are summarized in Table 2 and Fig. 6.

**Table 2:** Six differentially expressed proteins by PPI work

Uniprot ID	Symbol	Description	Subcellular location	Statistical Difference
D3DQH8	SPARC	Secreted protein acidic and rich in cysteine	Extracellular; Cytoplasmic	LNM/NC
P02790	HPX	Hemopexin	Extracellular	LNM/NC
P19320	VCAM1	Vascular cell adhesion protein 1	Nuclear	LNM/LVSI LVSI/NC
G3V0E5	TFRC	Transferrin receptor protein 1	Cytoplasmic	LVSI/NC
O75460	ERN1	Serine/threonine-protein kinase/endoribonuclease IRE1	Cytoplasmic; Nuclear	LNM/NC
Q9HDC9	APMAP	Adipocyte plasma membrane-associated protein	Cytoplasmic	LNM/NC LVSI/NC

### WGCNA

**Fig. 7:**
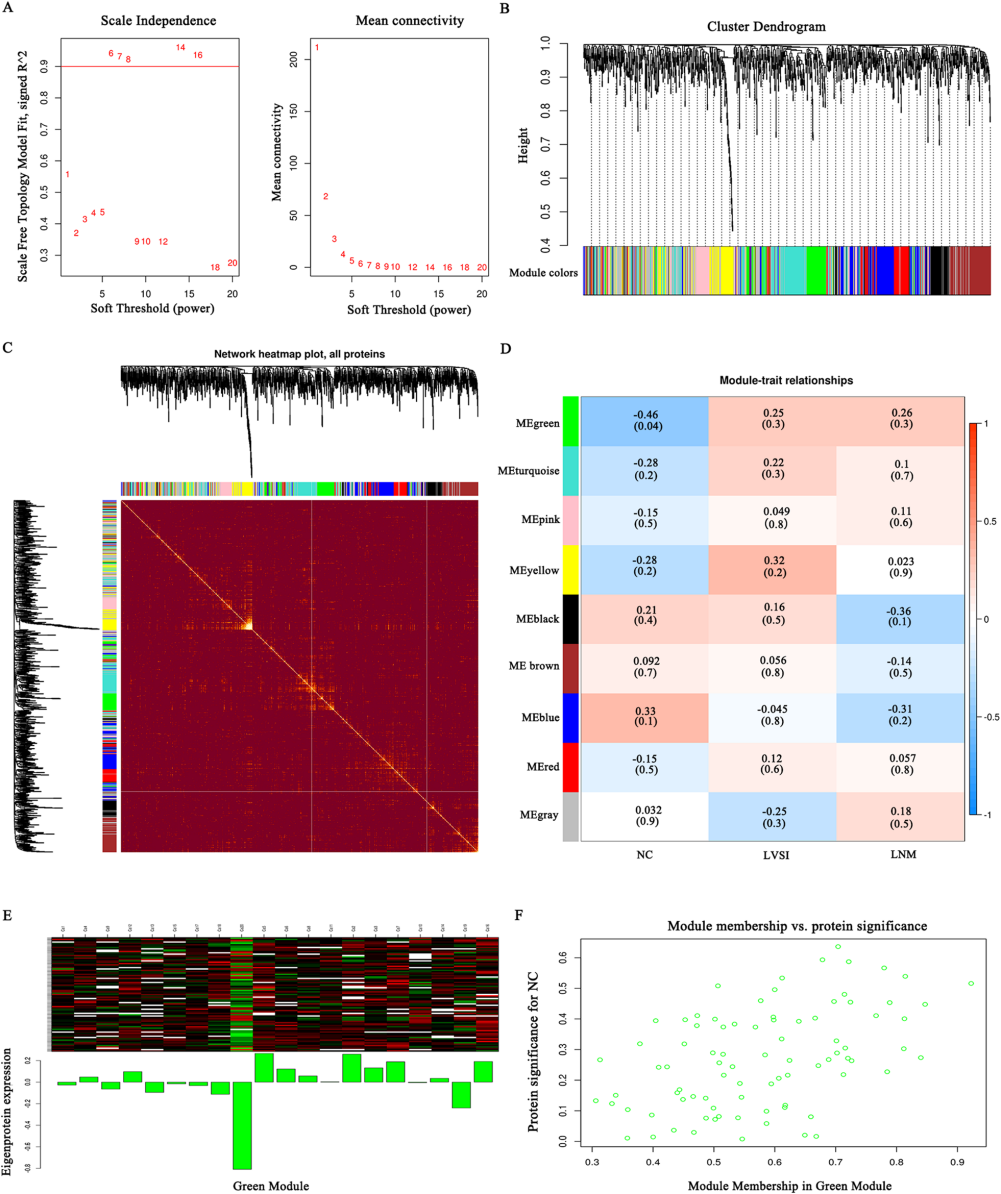
WGCNA of DEPs. **A** Scale-free fit metrics of the network topology obtained using soft threshold analysis. **B** Hierarchical clustering tree diagram. **C** Heatmap of the visualized gene network. The heatmap depicts the TOM between all DEPs in the analysis. Light colours indicate low overlap, and dark colours indicate high overlap. **D** A total of nine coexpression modules were constructed, and each colour represents one module of the protein coexpression network constructed by WGCNA. The top numbers in each cell represent Pearson r, and the bottom numbers represent P values. **E** Trend of green module protein expression. **F** The scatter distribution of gene significance and module membership

WGCNA is a method for analysing molecular expression patterns of multiple samples. It can cluster molecules with similar expression patterns and analyse the association between modules and specific traits or phenotypes. Therefore, WGCNA is widely used in the study of diseases and other traits. We performed WGCNA on these three groups of samples. Based on the optimal soft-power threshold β (Fig. 7A), the protein expression correlation coefficients were calculated. Then, protein hierarchical clustering trees and network heatmap plots were constructed to describe the different coexpression modules (Fig. 7B, C). A total of nine coexpression modules were constructed (Fig. 7D). Based on the criteria (Pearson r ≥ 0.4 or r ≤ − 0.4, P value ≤ 0.05), the green module of 84 proteins appeared statistical difference. The coexpressed protein network of the green module was visualized (Fig. 7E, F).

### Functional analysis of the green module from WGCNA

**Fig. 8:**
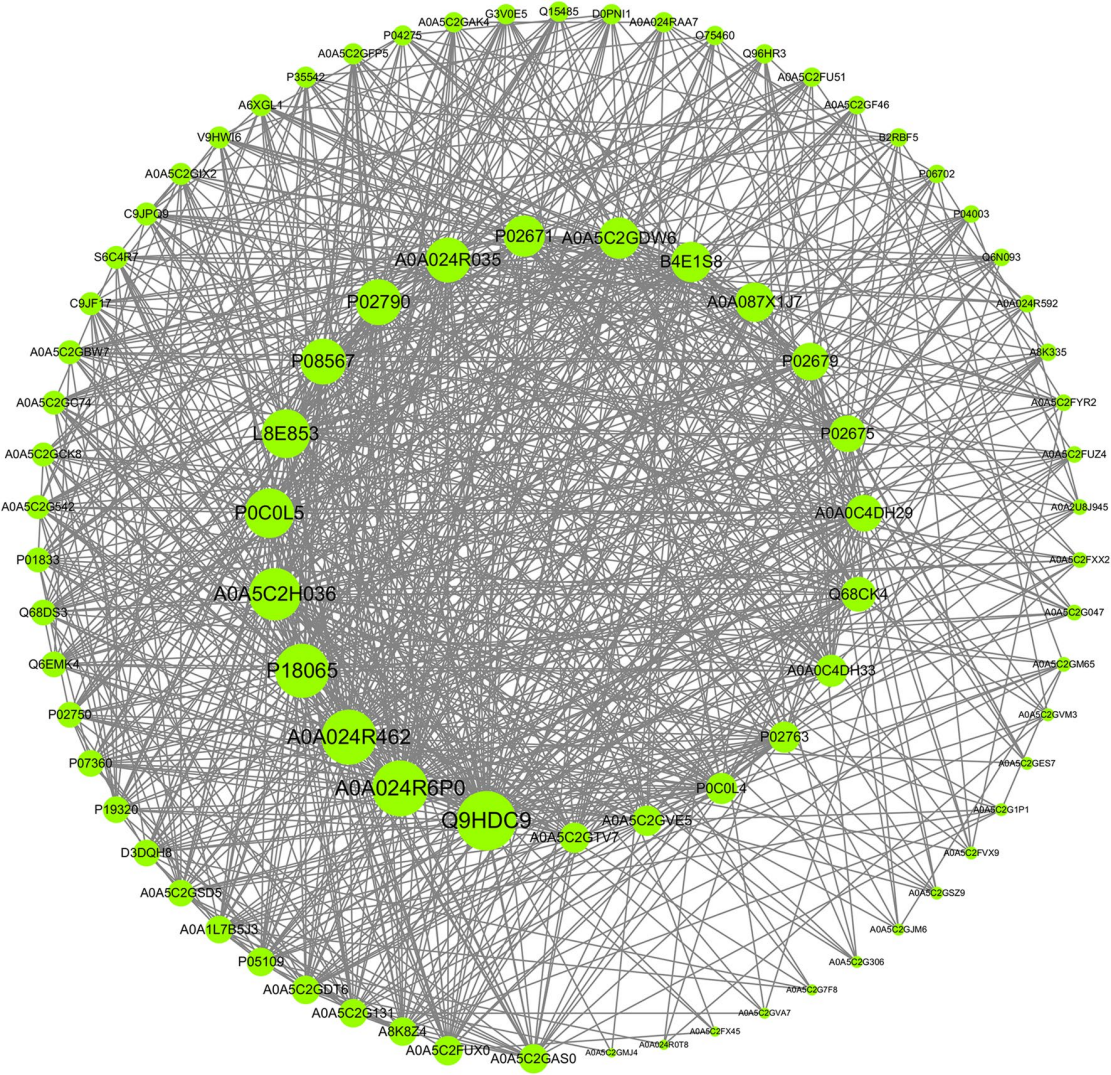
DEP coexpression network of the green module. The higher the number of connecting lines is, the larger the size of the node and the higher the importance in the protein‒protein interaction (PPI) network

**Fig. 9:**
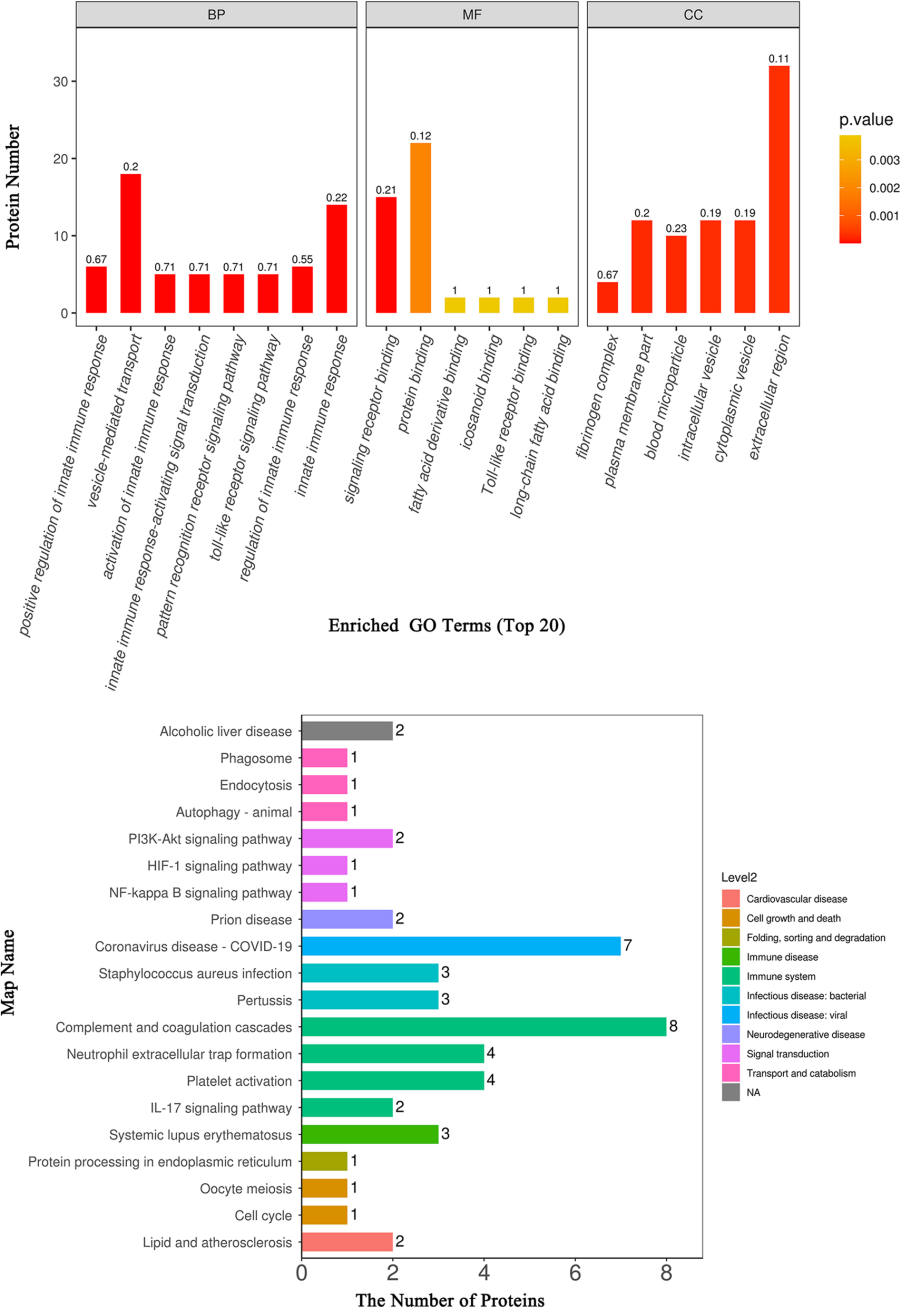
GO and KEGG pathway analysis of the proteins in the green module. **A** GO analysis. The BP, MF and CC categories are presented. **B** KEGG pathway analysis

To study the green module’s hub proteins and molecular mechanisms from a systematic perspective, we constructed a PPI network and performed GO and KEGG analysis. We identified hub proteins such as adipocyte plasma membrane-associated protein (APMAP, Q9HDC9), insulin-like growth factor-binding protein (IGF-BP2, P18065) and α-1-acid-glycoprotein 1 (ORM1, P02763). They are all summarized in Fig. 8. In addition, the GO enrichment analysis showed that vesicle-mediated transport and the innate immune response were important in the BP category, signalling receptor binding and protein binding were important in the MF category, and extracellular region was important in the CC category. KEGG enrichment analysis suggested that the green module DEPs were mainly enriched in complement and coagulation cascades, while other KEGG pathways, such as platelet activation, the PI3K-Akt signalling pathway and the HIF-1 signalling pathway, were also enriched (Fig. 9).

## Discussion

Disease stage and lymph node involvement are two of the most important prognostic factors in cervical cancer patients. According to the latest meta-analysis, the LNM incidence rate in the pelvic region ranged from 2% (stage IA2) to 14–36% (IB), 38–51% (IIA) and 47% (IIB), and in the para-aortic region, the LNM rate ranged from 2 to 5% (stage IB), 10–20% (IIA), 9% (IIB), 13–30% (III) and 50% (IV) [11]. Notably, these data were based on the 2009 FIGO staging system. Despite all the progress of imaging technologies such as TVUS/CT/MRI/PET, as well as the widely used SCC biomarker in squamous cell cervical cancer, the prediction rates are not robust enough to make a safe decision on whether to perform surgery in the pelvic or para-aortic region. Additionally, the mechanisms of LNM in cervical cancer are still not clear, and some studies have focused on changes in signalling pathways related to EMT, STAT3/p-STAT3, and immune escape [12–14]. Therefore, this study was designed to identify new candidate biomarkers and possible pathogenic mechanisms for cervical cancer. LVSI is a prerequisite and risk factor for LNM, and previous reports suggested that tumours with LVSI had a 9.3–12.0% LNM rate [15]. Therefore, LVSI was included as a separate study group in our study.

Our results show that the occurrence of LNM and LVSI is a process involving multiple systems and multiple signalling pathways. In addition to the complement and coagulation cascades we mentioned above, we found that the lipid and atherosclerosis pathways were significantly differentially enriched in the LNM vs. NC group as well as in the green module KEGG analysis. Considering these results and the results of the GO analysis, we propose that lipid metabolism may play an important role in lymph node metastasis. Some new published studies have focused on this topic and explored the regulatory mechanisms in cervical cancer [16, 17]. The HIF-1 signal transduction pathway changed greatly in the LVSI vs. NC group as well as in the green module, which was also proven by cytological and animal model experiments in some studies [18, 19]. In addition to this commonly studied pathway, we found some pathways worthy of more attention, including the phagosome and autophagy pathway; the modulation of this pathway in the context of hepatocellular carcinoma cell migration through EMT has only been reported in one study [20]. The ferroptosis pathway could promote the metastasis of cancer cells in melanoma, oesophageal squamous cell carcinoma, colon adenocarcinoma and prostate cancer [21–24]; however, its has not been well studies in the context of cervical cancer metastasis, so this will be the focus of our next study.

In total, six candidate biomarkers were screened out: SPARC, HPX, VCAM1, TFRC, ERN1 and APMAP. Among them, four proteins (SPARC, HPX, ERN, APMAP) were significantly upregulated in the LNM/NC group, three proteins (VCAM1, TFRC, APMAP) were upregulated in the LVSI/NC group, and one protein (APMAP) was upregulated in the LNM/LVSI group.

SPARC is a calcium-binding matricellular glycoprotein that is able to interact with various extracellular matrix macromolecules and regulate cell adhesion, proliferation and migration [25]. It has been widely reported to be associated with EMT in cervical cancer [26–28], and its methylation frequency increases with the severity of the underlying cervical lesion [29]. The serum level of SPARC was higher in cervical cancer patients than in healthy controls and CIN patients [28], and high expression of SPARC was correlated with the LNM of cervical carcinomas, which is consistent with the results of our DIA proteome sequencing study. However, we have yet to see its widespread application in clinical work.

VCAM-1 appears to be functionally important in the immune response and vascularization in high-grade cervical intraepithelial lesions and cervical cancer patients in several reports [30–32], and the study of its role as a molecular marker in the diagnosis of cervical diseases has been limited to histological and cytological specimens [31, 33, 34]. The detection of serum or plasma in patients with cervical disease has not been reported. TFRC was reported to be related to the prognosis of cervical cancer patients by participating in the JAK-STAT pathway and HIF-1 signalling pathway in some bioinformatics analysis articles [35, 36]. APMAP promotes the EMT and metastasis of cervical cancer cells by activating the Wnt/β-catenin pathway, and ERN1 is related to the apoptosis of cervical cancer cells [37, 38]. Unlike the molecules mentioned above, according to the results of our literature search, HPX has rarely been reported in the context of cervical disease. However, its diagnostic value has been proven in gallbladder carcinoma, hepatocellular carcinoma, breast cancer, prostate cancer [39–41], and so on. Therefore, we believe that these five protein molecules (including SPARC) have the potential to be plasma biomarkers in cervical cancer patients.

## Conclusions

In this study, we assessed the whole-protein profiles to identify factors related to LVSI and LNM in cervical cancer through plasma DIA-based quantitative proteomics. Through the analysis of 79 DEPs identified by bioinformatics methods, we found six new candidate biomarkers and a series of pathways (such as the phagosome and autophagy pathway) that are involved in the whole pathogenic process, and further functional studies and validation studies would be worthwhile.

### Supplementary Information

**Additional file 1: Figure S1.** The top nine DEPs with a significant difference between every two groups. **Figure S2.** Subcellular localization and domain enrichment analysis of DEPs between the LNM and NC groups. **Figure S3.** GO and KEGG pathway analysis of DEPs between the LNM and NC groups. **Figure S4.** Subcellular localization and domain enrichment analysis of DEPs between the LVSI and NC groups. **Figure S5.** GO and KEGG pathway analysis of DEPs between the LVSI and NC groups. **Figure S6.** Subcellular localization and domain enrichment analysis of DEPs between the LNM and LVSI groups. **Figure S7.** GO and KEGG pathway analysis of DEPs between the LNM and LVSI groups.

## Data Availability

The data used and/or analysis during the current study are available from the corresponding author on reasonable request.
