# Supplementary figures and images for: New mechanisms and biomarkers of lymph node metastasis in cervical cancer: reflections from plasma proteomics
Source: Clin Proteomics. 2023 Sep 9;20:35. doi: 10.1186/s12014-023-09427-8 (PMC10492398; doi:10.1186/s12014-023-09427-8)

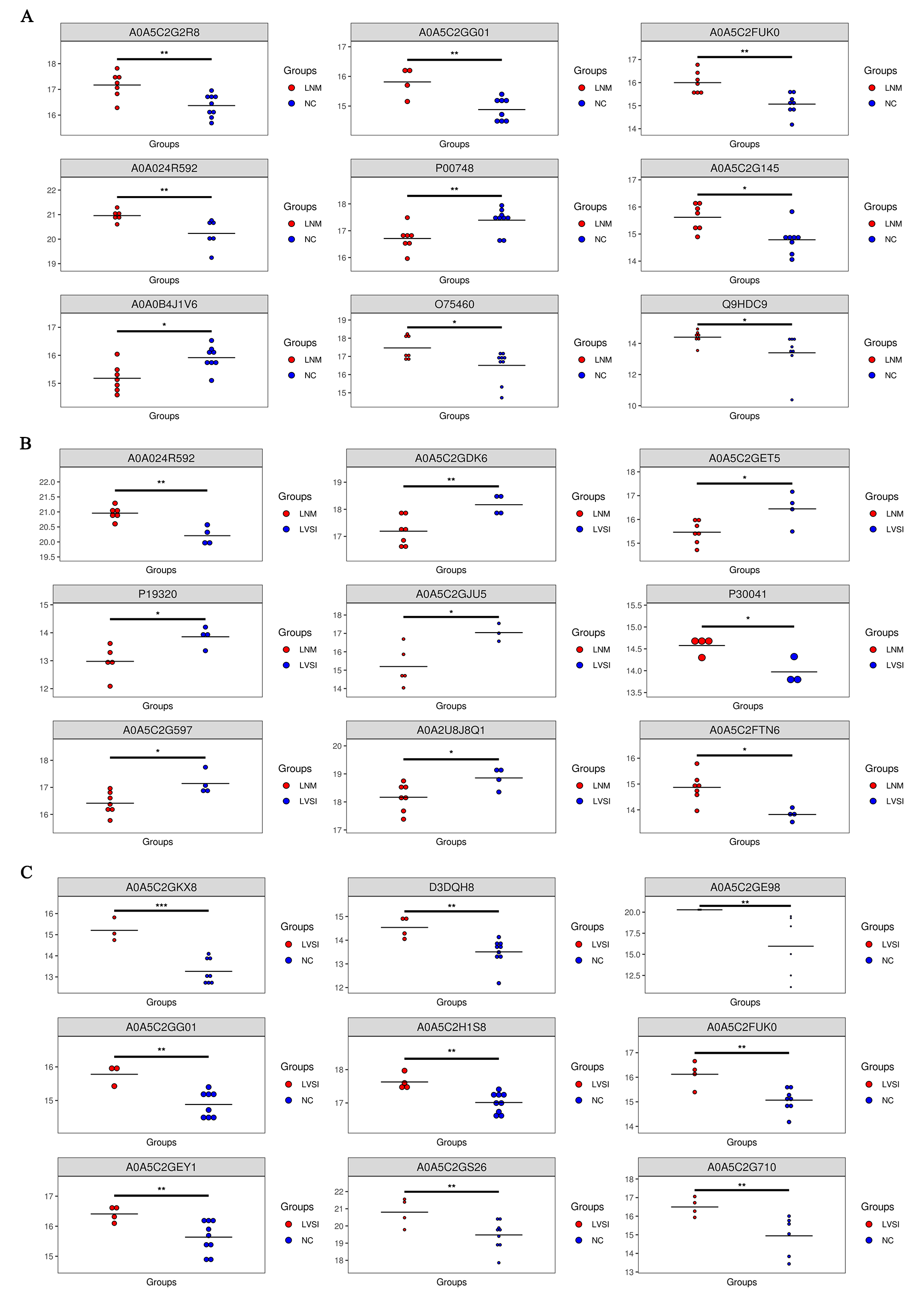

Supplement: Supplementary file 1 — Additional file 1: Figure S1. The top nine DEPs with a significant difference between every two groups. Figure S2. Subcellular localization and domain enrichment analysis of DEPs between the LNM and NC groups. Figure S3. GO and KEGG pathway analysis of DEPs between the LNM and NC groups. Figure S4. Subcellular localization and domain enrichment analysis of DEPs between the LVSI and NC groups. Figure S5. GO and KEGG pathway analysis of DEPs between the LVSI and NC groups. Figure S6. Subcellular localization and domain enrichment analysis of DEPs between the LNM and LVSI groups. Figure S7. GO and KEGG pathway analysis of DEPs between the LNM and LVSI groups. [file 12014_2023_9427_MOESM1_ESM.zip › Supplementary Materials/Supplementary Figure 1.tif]

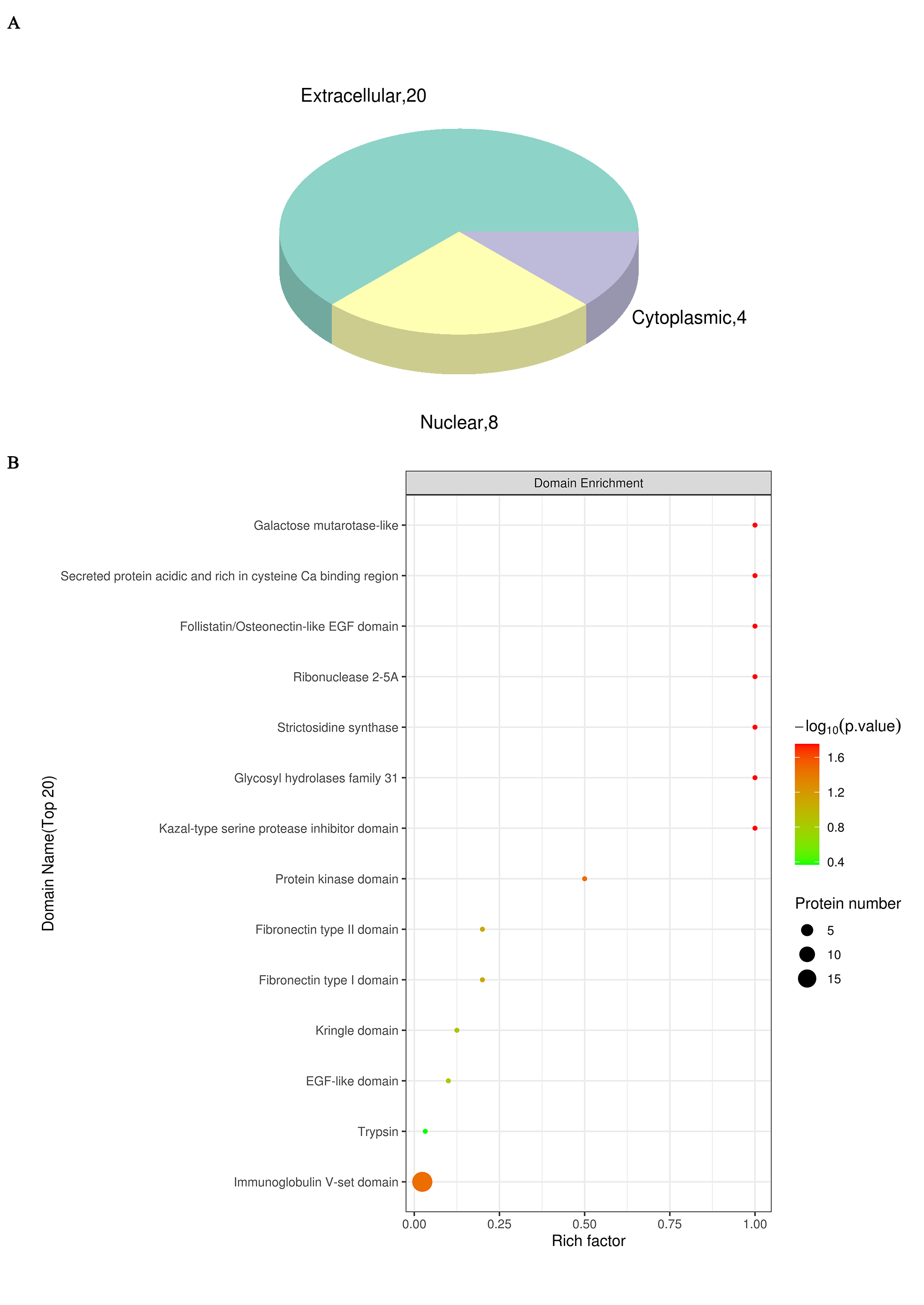

Supplement: Supplementary file 1 — Additional file 1: Figure S1. The top nine DEPs with a significant difference between every two groups. Figure S2. Subcellular localization and domain enrichment analysis of DEPs between the LNM and NC groups. Figure S3. GO and KEGG pathway analysis of DEPs between the LNM and NC groups. Figure S4. Subcellular localization and domain enrichment analysis of DEPs between the LVSI and NC groups. Figure S5. GO and KEGG pathway analysis of DEPs between the LVSI and NC groups. Figure S6. Subcellular localization and domain enrichment analysis of DEPs between the LNM and LVSI groups. Figure S7. GO and KEGG pathway analysis of DEPs between the LNM and LVSI groups. [file 12014_2023_9427_MOESM1_ESM.zip › Supplementary Materials/Supplementary Figure 2 LNM vs NC.tif]

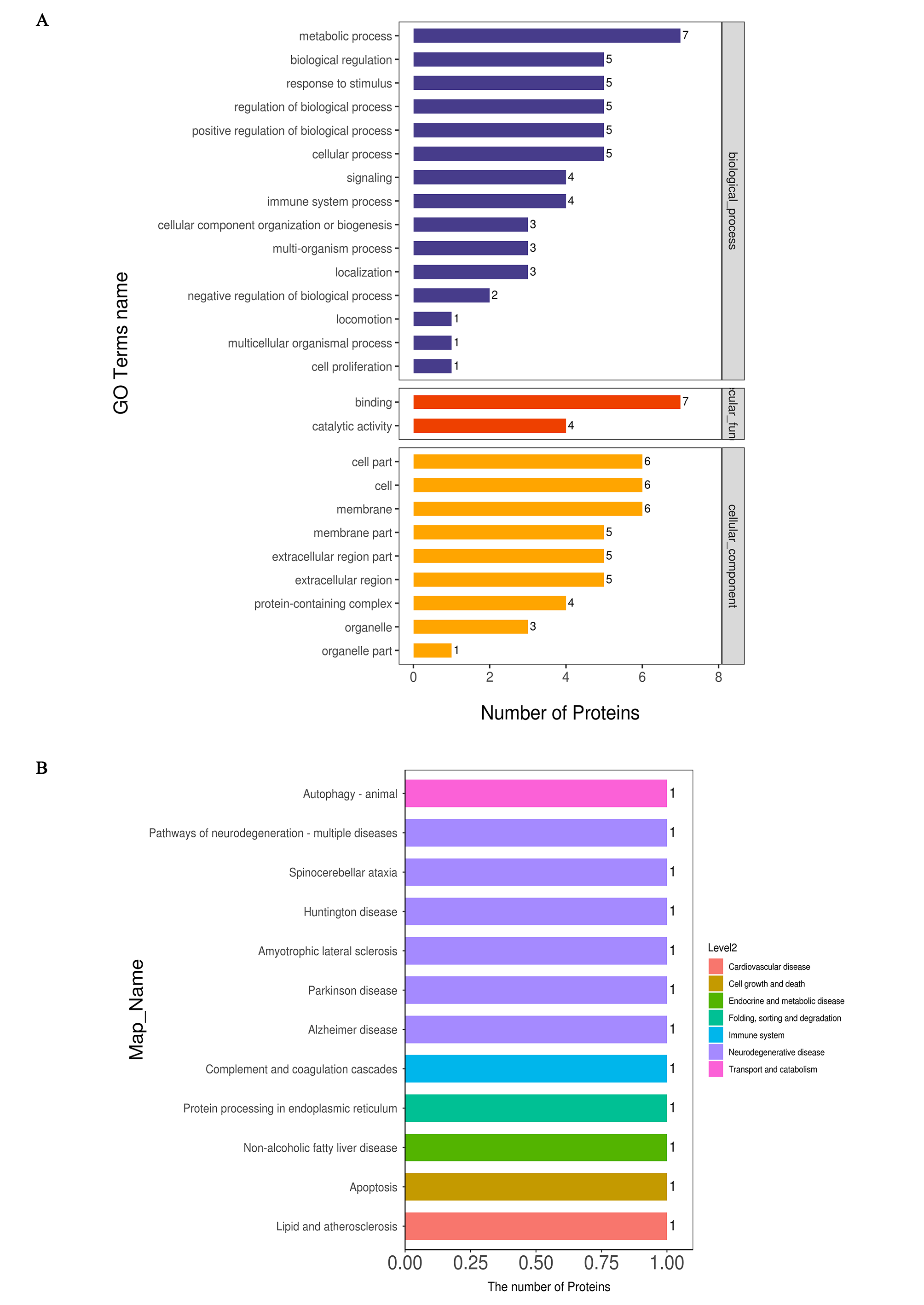

Supplement: Supplementary file 1 — Additional file 1: Figure S1. The top nine DEPs with a significant difference between every two groups. Figure S2. Subcellular localization and domain enrichment analysis of DEPs between the LNM and NC groups. Figure S3. GO and KEGG pathway analysis of DEPs between the LNM and NC groups. Figure S4. Subcellular localization and domain enrichment analysis of DEPs between the LVSI and NC groups. Figure S5. GO and KEGG pathway analysis of DEPs between the LVSI and NC groups. Figure S6. Subcellular localization and domain enrichment analysis of DEPs between the LNM and LVSI groups. Figure S7. GO and KEGG pathway analysis of DEPs between the LNM and LVSI groups. [file 12014_2023_9427_MOESM1_ESM.zip › Supplementary Materials/Supplementary Figure 3 LNM vs NC.tif]

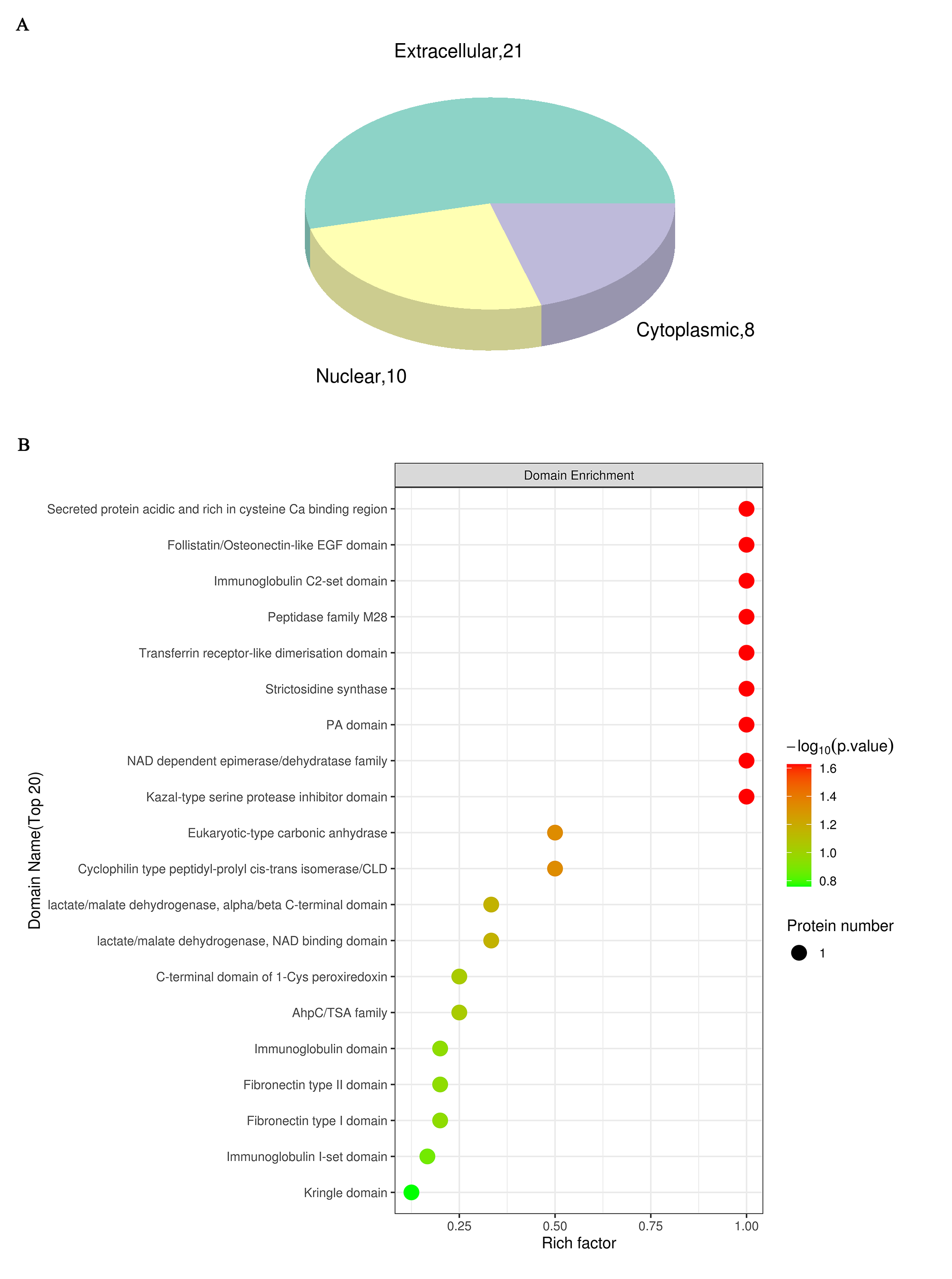

Supplement: Supplementary file 1 — Additional file 1: Figure S1. The top nine DEPs with a significant difference between every two groups. Figure S2. Subcellular localization and domain enrichment analysis of DEPs between the LNM and NC groups. Figure S3. GO and KEGG pathway analysis of DEPs between the LNM and NC groups. Figure S4. Subcellular localization and domain enrichment analysis of DEPs between the LVSI and NC groups. Figure S5. GO and KEGG pathway analysis of DEPs between the LVSI and NC groups. Figure S6. Subcellular localization and domain enrichment analysis of DEPs between the LNM and LVSI groups. Figure S7. GO and KEGG pathway analysis of DEPs between the LNM and LVSI groups. [file 12014_2023_9427_MOESM1_ESM.zip › Supplementary Materials/Supplementary Figure 4 LVSI vs NC.tif]

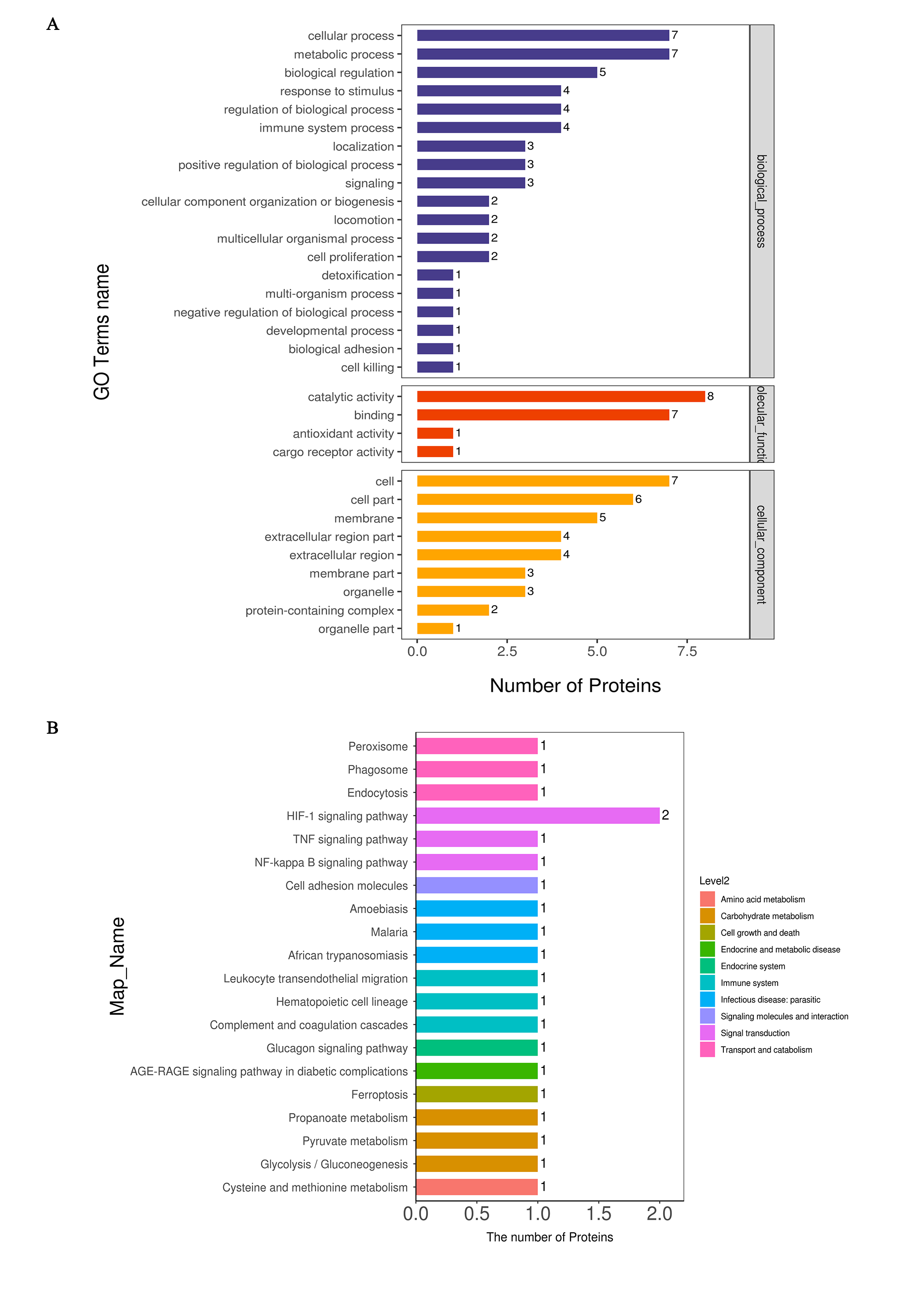

Supplement: Supplementary file 1 — Additional file 1: Figure S1. The top nine DEPs with a significant difference between every two groups. Figure S2. Subcellular localization and domain enrichment analysis of DEPs between the LNM and NC groups. Figure S3. GO and KEGG pathway analysis of DEPs between the LNM and NC groups. Figure S4. Subcellular localization and domain enrichment analysis of DEPs between the LVSI and NC groups. Figure S5. GO and KEGG pathway analysis of DEPs between the LVSI and NC groups. Figure S6. Subcellular localization and domain enrichment analysis of DEPs between the LNM and LVSI groups. Figure S7. GO and KEGG pathway analysis of DEPs between the LNM and LVSI groups. [file 12014_2023_9427_MOESM1_ESM.zip › Supplementary Materials/Supplementary Figure 5 LVSI vs NC.tif]

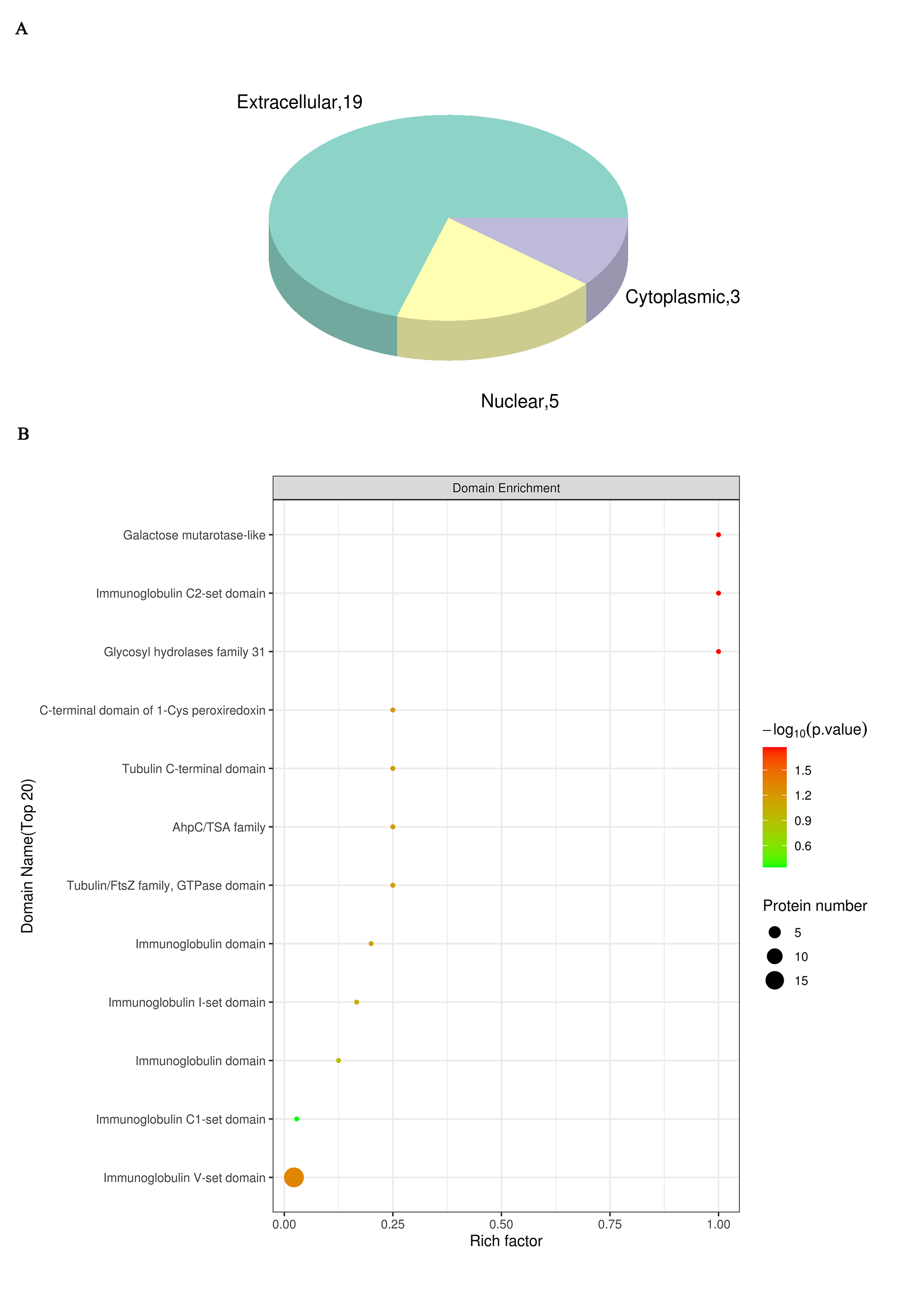

Supplement: Supplementary file 1 — Additional file 1: Figure S1. The top nine DEPs with a significant difference between every two groups. Figure S2. Subcellular localization and domain enrichment analysis of DEPs between the LNM and NC groups. Figure S3. GO and KEGG pathway analysis of DEPs between the LNM and NC groups. Figure S4. Subcellular localization and domain enrichment analysis of DEPs between the LVSI and NC groups. Figure S5. GO and KEGG pathway analysis of DEPs between the LVSI and NC groups. Figure S6. Subcellular localization and domain enrichment analysis of DEPs between the LNM and LVSI groups. Figure S7. GO and KEGG pathway analysis of DEPs between the LNM and LVSI groups. [file 12014_2023_9427_MOESM1_ESM.zip › Supplementary Materials/Supplementary Figure 6 LNM vs LVSI.tif]

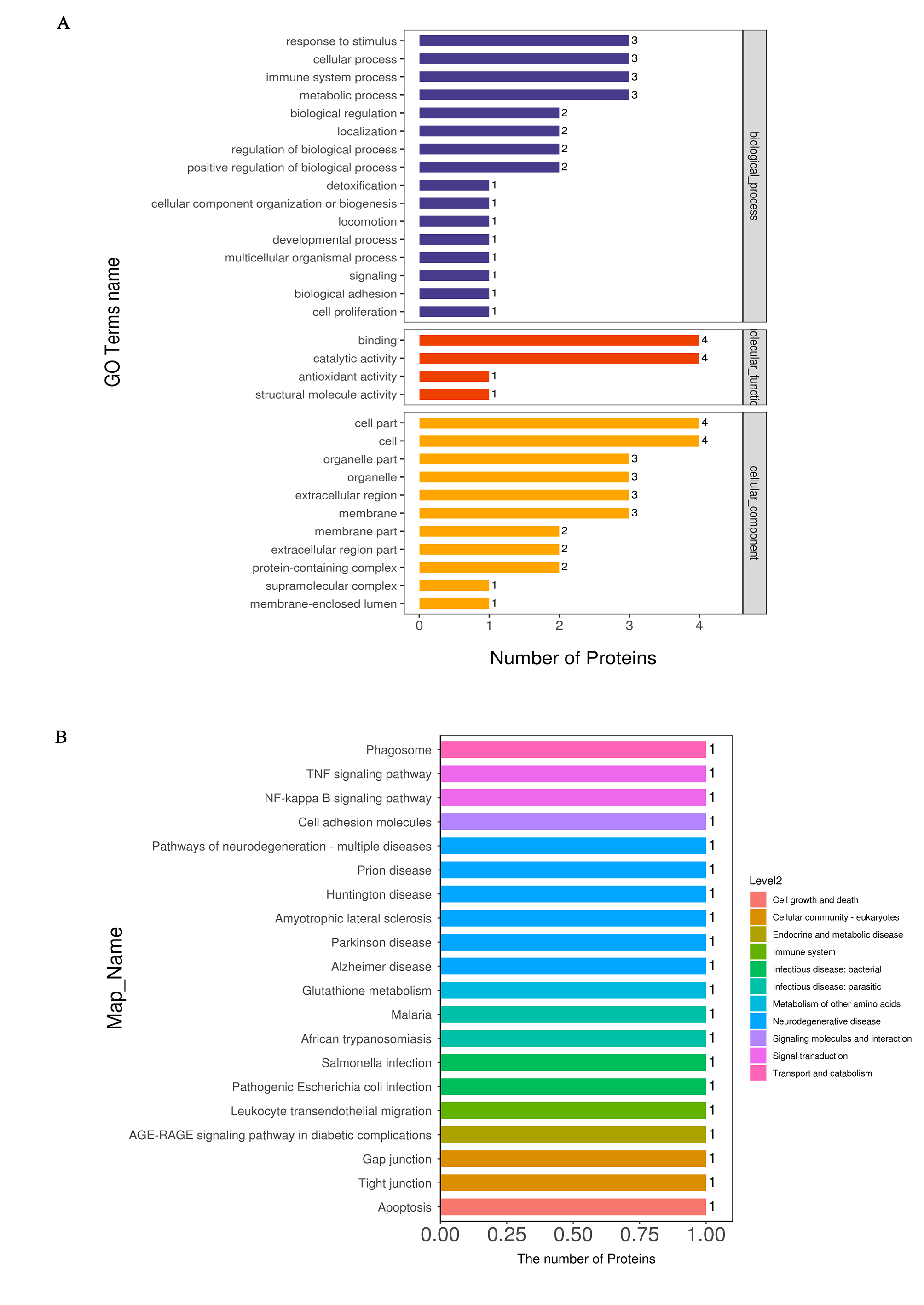

Supplement: Supplementary file 1 — Additional file 1: Figure S1. The top nine DEPs with a significant difference between every two groups. Figure S2. Subcellular localization and domain enrichment analysis of DEPs between the LNM and NC groups. Figure S3. GO and KEGG pathway analysis of DEPs between the LNM and NC groups. Figure S4. Subcellular localization and domain enrichment analysis of DEPs between the LVSI and NC groups. Figure S5. GO and KEGG pathway analysis of DEPs between the LVSI and NC groups. Figure S6. Subcellular localization and domain enrichment analysis of DEPs between the LNM and LVSI groups. Figure S7. GO and KEGG pathway analysis of DEPs between the LNM and LVSI groups. [file 12014_2023_9427_MOESM1_ESM.zip › Supplementary Materials/Supplementary Figure 7 LNM vs LVSI.tif]
